# Characterizing Digital Mindfulness Intervention Utilization and Weekly Assessments in Caregivers of Persons Living With Dementia: Secondary Analysis of a Randomized Controlled Trial

**DOI:** 10.2196/83618

**Published:** 2026-07-21

**Authors:** Michael P Williams, Darby M Simon, Morgan Seward, Raquel G Tatar, Jennifer Huberty, Ana-Maria Vranceanu, Evan Plys

**Affiliations:** 1 Center for Health Outcomes and Interdisciplinary Research, Massachusetts General Hospital, 1 Bowdoin Square, Suite 100, Boston, MA, 02114, United States, 1 585-313-3912; 2 Department of Psychiatry, Harvard Medical School, Boston, MA, United States; 3 Healthy Minds Innovation, Madison, WI, United States; 4 Department of Medicine, University of Texas Health, San Antonio, TX, United States

**Keywords:** caregivers, mindfulness, stress, dementia, digital health, utilization

## Abstract

**Background:**

Caregivers of persons living with dementia are at increased risk of reporting high stress. Mindfulness-based interventions (MBIs) teach caregivers mindfulness skills and are effective at reducing stress. Digital MBIs are a feasible way to improve access to MBIs for caregivers of persons living with dementia. Yet, caregiver improvement with digital MBI utilization is less defined in the literature.

**Objective:**

The goal of this secondary data analysis was to characterize weekly mindfulness and stress ratings among caregivers of persons living with dementia and to examine how digital MBI utilization impacted these ratings throughout 12 weeks of a feasibility trial.

**Methods:**

Participants were eligible for this secondary analysis if they were randomized to the digital MBI condition (Healthy Minds Program for Caregivers [HMP-C], n=46) and completed weekly ratings over the 12-week trial. At baseline and at the end of each week of the trial, participants rated their mindfulness and stress in the past week from 0 to 10. Weekly HMP-C utilization was defined as the time spent using HMP-C in the week prior to the weekly ratings. Descriptive statistics and visualizations were used to characterize mindfulness and stress ratings. Generalized linear mixed models were used to estimate the effect of mindfulness on stress and the effect of HMP-C utilization on stress and mindfulness throughout the trial (α=.05).

**Results:**

Baseline mindfulness and stress ratings were 4 (IQR 3-6, range 0‐8) and 7 (IQR 6.5-8, range 4‐10), respectively. Stress had the greatest decrease between baseline and week 3 (−2 points on average), whereas mindfulness had the greatest increase between baseline and week 4 (+2.5 points on average). There was a significant fixed effect of baseline mindfulness on baseline stress (β=.5, *P*<.001), with a significant interaction between mindfulness and study week (β=.05, *P*=.001), suggesting that this relationship was attenuated over time. There was variability in baseline stress (τ_00_=2.04) and the relationship between mindfulness and stress (τ_₁₁_=0.08), with a high correlation (ρ_01_=0.86), suggesting that those with high baseline stress benefited most from increases in mindfulness. For every 10 minutes of HMP-C utilization between baseline and week 1, mindfulness and stress ratings were 0.14 points higher (*P*<.001) and 0.14 points lower (*P*<.001), respectively. Despite a significant interaction between HMP-C utilization and study week in both models, the effect size was small (mindfulness: β=.02, *P*<.001; stress: β=.01, *P*=.013), suggesting that this relationship was sustained over time.

**Conclusions:**

Mindfulness and stress changed mostly during the first 3 to 4 weeks of the trial. During this time of mindfulness skill acquisition, mindfulness and stress were most significantly negatively associated, especially among those with high baseline stress. The consistent relationship between HMP-C utilization and mindfulness and stress suggests that continued use of HMP-C may be helpful for skill maintenance.

## Introduction

There are approximately 53 million informal caregivers of persons living with dementia in the United States [1]. Caregivers of persons living with dementia are at higher risk for poor mental and physical health outcomes compared with age-matched noncaregiver peers [2]. Notably, caregivers of persons living with dementia experience high levels of stress [3], defined as a state of worry or mental tension caused by difficult situations [4]. Reducing stress provides an opportunity to improve caregiver well-being, and approximately half of caregivers report a desire for stress management interventions [5]. Together, there is a need and demand for stress reduction interventions among caregivers of persons living with dementia.

Mindfulness-based interventions (MBIs) are an effective nonpharmacological treatment for caregiver stress [6]. Mindfulness can be conceptualized as both a *trait*, or one’s predisposition to be mindful in daily life, and a *state* that is achieved while practicing mindful meditation [7]. A framework proposed by Dahl et al [8] posits that state mindfulness meditation practices consist of skills that can be trained and developed (awareness, connection, insight, and purpose). MBIs primarily teach participants mindfulness skills as a coping strategy to manage everyday stressors. Mindfulness skills center on nonjudgmentally observing thoughts, feelings, and behaviors in the present moment [9]. The continual practice of state mindfulness through these skills increases trait mindfulness over time [7]. Both state and trait mindfulness are associated with lower perceived stress across multiple populations [10-12], including caregivers of persons living with dementia [13-15].

Although MBIs can reduce caregiver stress, caregivers often experience barriers to accessing these interventions in outpatient settings. For example, caregivers may experience limited access to respite care [16], limited free time [17], and resistance to leaving their care recipient due to guilt or fear [18], all of which interfere with help-seeking. Even when interventions are offered remotely, they are often synchronous and do not allow for additional flexibility when acute caregiving responsibilities arise. Digital MBIs provide an opportunity to overcome these barriers and increase access to MBIs for caregivers [19-22]. Digital MBIs are often delivered via mobile phone apps, are free or inexpensive, and can be accessed at any time, promoting flexibility in use, which may be particularly beneficial for those in the caregiving role. Digital MBIs are feasible and acceptable among caregivers of persons living with dementia in pilot trials [23-25] and align with a growing trend of technology acceptance among caregivers [26,27].

Although digital MBIs show promise for reducing stress among caregivers, the trajectory of skill acquisition and perceived stress reduction during participation in digital MBIs is rarely studied. According to Dahl and colleagues’ [8] conceptualization of mindfulness as a series of skills to be learned, mindfulness is not expected to increase indefinitely, but rather to be learned (ie, skill acquisition), applied (ie, used as a state in stressful situations), and maintained (ie, developed as a trait across situations). A more granular understanding of the trajectory of skill acquisition and perceived stress reduction could help the scalability of interventions in a real-world context (eg, recommended usage, benchmarks for progress). This information may be particularly beneficial for self-directed interventions, such as digital MBIs, which lack regular monitoring of progress by a trained clinician.

This study aimed to elucidate the relationships among caregiver mindfulness, stress, and utilization of a digital MBI. This study is an exploratory secondary analysis of a feasibility pilot randomized controlled trial (RCT) of Healthy Minds Program for Caregivers (HMP-C), a digital MBI tailored to caregivers of persons living with dementia [25]. The primary pilot RCT reported findings primarily in the domains of feasibility, acceptability, treatment satisfaction, and adherence. The distinct aims of this study were 3-fold: (1) to characterize the trends of caregiver mindfulness and stress across the 12-week duration of the primary study, (2) to characterize the relationships between participants’ weekly mindfulness and stress ratings, and (3) to explore how participants’ utilization of the digital MBI is associated with their weekly mindfulness and stress ratings across the study duration.

## Methods

### Study Design

This study is an exploratory secondary data analysis of a single-blind pilot RCT (National Institutes of Health Stage Model, Stage 1b) of HMP-C, a digital MBI tailored for caregivers of persons living with dementia.

### Ethical Considerations

This trial is registered with ClinicalTrials.gov (NCT05732038), and all study procedures were approved by the affiliated institutional review board (2022P001601) [25,28]. Informed consent was collected electronically. A member of the study staff was present to answer any questions. Participants received a total of US $50 for completing the postassessment and follow-up assessments. The privacy and confidentiality of all participant data were maintained.

### Participants

The primary single-blind feasibility pilot RCT recruited informal adult caregivers of a known person living with Alzheimer disease or a related dementia reporting elevated levels of perceived stress. Recruitment was carried out through flyers and online advertisements. Interested caregivers referred themselves via an online link included in the recruitment materials. Here, they completed a brief eligibility questionnaire (verified by a phone call with study staff) based on the following inclusion criteria: (1) >18 years old, (2) English literacy, (3) self-identify as an informal caregiver, (4) Perceived Stress Scale-10 score of ≥6 [29,30], (5) willing to be randomized, and (6) care recipient rated >1 on the Functional Assessment Staging Tool [31]. Exclusion criteria included (1) any planned change in psychotropic pharmacological treatment for the duration of the study, (2) use of any consumer-based mindfulness meditation app for more than 60 minutes per month in the past 6 months, (3) current participation in a meditation program, (4) major illness anticipated to worsen dramatically or require surgery in the next 20 weeks, (5) active treatment for cancer (eg, chemotherapy, radiation), (6) placement of the care recipient in a nursing home, (7) involvement in another clinical trial for caregivers, and (8) 4+ errors on the Short Portable Mental Status Questionnaire (ie, for caregivers older than 65 years of age) [32]. The primary feasibility RCT analyzed 90 participants across the HMP-C intervention (n=46) and a control (n=44) condition [25]. The present secondary analysis utilized participants in the intervention condition only, as the control condition did not teach mindfulness skills.

### Intervention

HMP-C is a digital MBI tailored for caregivers of persons living with dementia, based on content from HMP, a publicly available mobile app [25,33]. HMP consists of written and guided listening content that aims to help users develop the following mindfulness skills: (1) awareness (eg, mindfulness, attention, self-awareness), (2) connection (eg, appreciation, kindness, compassion), (3) insight (eg, self-inquiry, self-knowledge, transcending the self), and (4) purpose (eg, purpose clarity, embodying values, finding meaning) [8,33]. Based on the Dahl et al [8] framework, these skills are taught to listeners through educational content based on the science of well-being and procedural guided mindfulness practices [25].

### Primary Pilot RCT Procedures

Participants were directed to use the app for at least 10 minutes per day, or 70 minutes per week, for 12 weeks. Participants were reminded each week by text message to use their respective phone app for the prescribed amount of time. App utilization data were reviewed weekly by study staff, and participants who did not use at least 75% of their recommended amount in the past week were sent an additional standardized reminder message. Participants who did not complete their recommended utilization for 2 consecutive weeks were called by a study staff member to problem-solve potential barriers to adherence. Throughout the 12-week trial period, participants were emailed a REDCap (Research Electronic Data Capture) link to weekly single-item stress and mindfulness assessments. Participants who completed all assessments were offered a monetary incentive of up to US $50.

### Measures

#### Weekly Mindfulness and Stress Assessments

At the end of each trial week, participants were asked to self-report perceived levels of mindfulness and stress from the prior week on single-item assessments, with scores ranging from 0 to 10. These assessments were also administered at baseline for a total of 13 weekly assessments (ie, baseline and 12 trial weeks). The stress assessment asked, “During the past week, how would you rate your stress on a scale from 0 to 10? (0=not at all; 10=extremely stressed).” The mindfulness assessment asked, “During the past week, how would you rate your mindfulness on a scale from 0 to 10? (0=not at all; 10=extremely mindful).” Mindfulness and stress among caregivers are multifaceted constructs, and several sources may contribute to stress among caregivers of persons living with dementia. The single-item measures utilized in this study represent broad, subjective ratings of mindfulness and stress regardless of perceived source.

#### Weekly HMP-C Utilization

Weekly HMP-C app utilization was defined as a continuous measure representing the sum of minutes of HMP-C utilization in the 7 days prior to each participant’s completion of their weekly mindfulness and stress assessments. These data were automatically captured through the HMP-C app.

### Statistical Analysis

#### Overview

All statistical analyses were conducted using R (R Foundation for Statistical Computing) [34]. Four participants were removed from the data prior to analysis due to missing data. Specifically, 2 participants completed no weekly stress or mindfulness assessments, and 2 participants completed several weekly assessments in batches (eg, completing 2 or more weekly assessments at the same time). The final analytic dataset used in this study comprised the remaining 42 participants who received HMP-C. Descriptive statistics were used to describe participant characteristics for the 42 eligible participants included in this analysis. Statistical approaches relevant to each aim are detailed below. Consistent with study aims outlined below, we analyzed completers only. An α of .05 was used for all statistical tests.

#### Aim 1: Characterize Stress and Mindfulness Throughout the Study

Weekly stress and mindfulness assessments were characterized using descriptive statistics, including the median, minimum, maximum, and IQR. Further, we described change scores for the study variables throughout the trial.

#### Aim 2: Characterize the Relationship Between Weekly Mindfulness and Stress Ratings Throughout the Study Duration

Generalized linear mixed models (GLMMs) were used to assess the relationship between mindfulness and stress throughout the course of the 12-week trial period. Mindfulness was mean-centered to improve interpretation. This model also included an interaction term for mindfulness and time (study week) to assess how the effect of mindfulness on stress changed throughout the study duration. This model included a random intercept and a random slope for mindfulness to account for individual differences in baseline stress and the effect of mindfulness on stress. Random and fixed effects are reported for the effect of weekly mindfulness ratings on weekly stress ratings. Additionally, we visualized the Pearson correlation coefficient between weekly mindfulness and stress ratings. The final reported model was assessed for the following model assumptions: (1) linearity of fixed effects, (2) residual distribution, (3) outliers, (4) autocorrelation, (5) dispersion, (6) link function specification, and (7) zero inflation. No major deviations were detected for any of the aforementioned criteria. A power analysis was conducted using a simulation-based approach (1000 replications) and the observed effect size for the interaction term.

#### Aim 3: Explore How HMP-C Utilization Is Associated With Weekly Mindfulness and Stress Ratings Throughout the Study Duration

GLMMs were used to test the association between HMP-C utilization and weekly mindfulness and stress ratings. Both models included an interaction term between HMP-C utilization and time (study week) to assess how these effects changed throughout the study duration. Initially, models were specified to include a random slope and random intercept, as in Aim 2. However, both models in Aim 3 exhibited a singular fit, indicating that the variance of the random slope was approximately zero. This is common in datasets with small-to-medium sample sizes [35]. Due to the inability to estimate a random slope, we reported models that only included a random intercept, which aligns with guidelines for building GLMMs [35]. HMP-C utilization was mean-centered for improved interpretation, and study week was centered at week 1 (as opposed to baseline), as no participants utilized HMP-C prior to the baseline assessment. Since we are interested in how past-week HMP-C utilization is associated with stress and mindfulness, centering study week at week 1 allows for a week’s worth of HMP-C utilization to transpire before estimating that association. Random and fixed effects are reported for the effect of HMP-C utilization on weekly mindfulness and stress ratings. The final reported model was assessed for the following model assumptions: (1) linearity of fixed effects, (2) residual distribution, (3) outliers, (4) autocorrelation, (5) dispersion, (6) link function specification, and (7) zero inflation. No major deviations were detected for any of the aforementioned criteria. Power analyses were conducted for both models using a simulation-based approach (1000 replications) and the observed effect size for the interaction term.

## Results

### Participants

Table 1 outlines participant characteristics at baseline for the 42 eligible caregivers included in this study. The average age was 55 (SD 14) years. Most participants were female by sex (n=39, 93%) and gender identity (n=38, 90%). Most participants were White (n=34, 81%). Most participants were married and living with their partner (n=28, 67%). Participants in this study used HMP-C for a median of 70 (IQR 41.7-102.2) minutes per week.

**Table 1.** Participant characteristics (N=42).

Characteristic	Value
Age (y), mean (SD)	55 (14)
Sex, n (%)	
Female	39 (93)
Male	3 (7.1)
Gender identity, n (%)	
Man	3 (7.1)
Woman	38 (90)
Prefer not to say	1 (2.4)
Race, n (%)	
Asian	1 (2.4)
Black/African American	4 (9.5)
More than one race	2 (4.8)
White	34 (81)
Choose not to answer	1 (2.4)
Ethnicity, n (%)	
Hispanic or Latino/Latina	3 (7.1)
Not Hispanic or Latino/Latina	38 (90)
Choose not to answer	1 (2.4)
Education, n (%)	
Completed high school or GED^a^	1 (2.4)
Some college	5 (12)
Associate degree	3 (7.1)
Completed 4 years of college	11 (26)
Graduate/professional degree	20 (48)
Trade/vocational training	2 (4.8)
Household income (US $), n (%)	
Less than 15,000	2 (4.8)
15,000 to 29,999	1 (2.4)
30,000 to 49,999	5 (12)
50,000 to 69,999	3 (7.1)
70,000 to 99,999	9 (21)
100,000 or more	17 (40)
Choose not to answer	5 (12)
Marital status, n (%)	
Married/partnered, living together	28 (67)
Separated/divorced	7 (17)
Single, never married	6 (14)
Choose not to answer	1 (2.4)

a GED: General Educational Development.

### Aim 1

**Figure 1.**
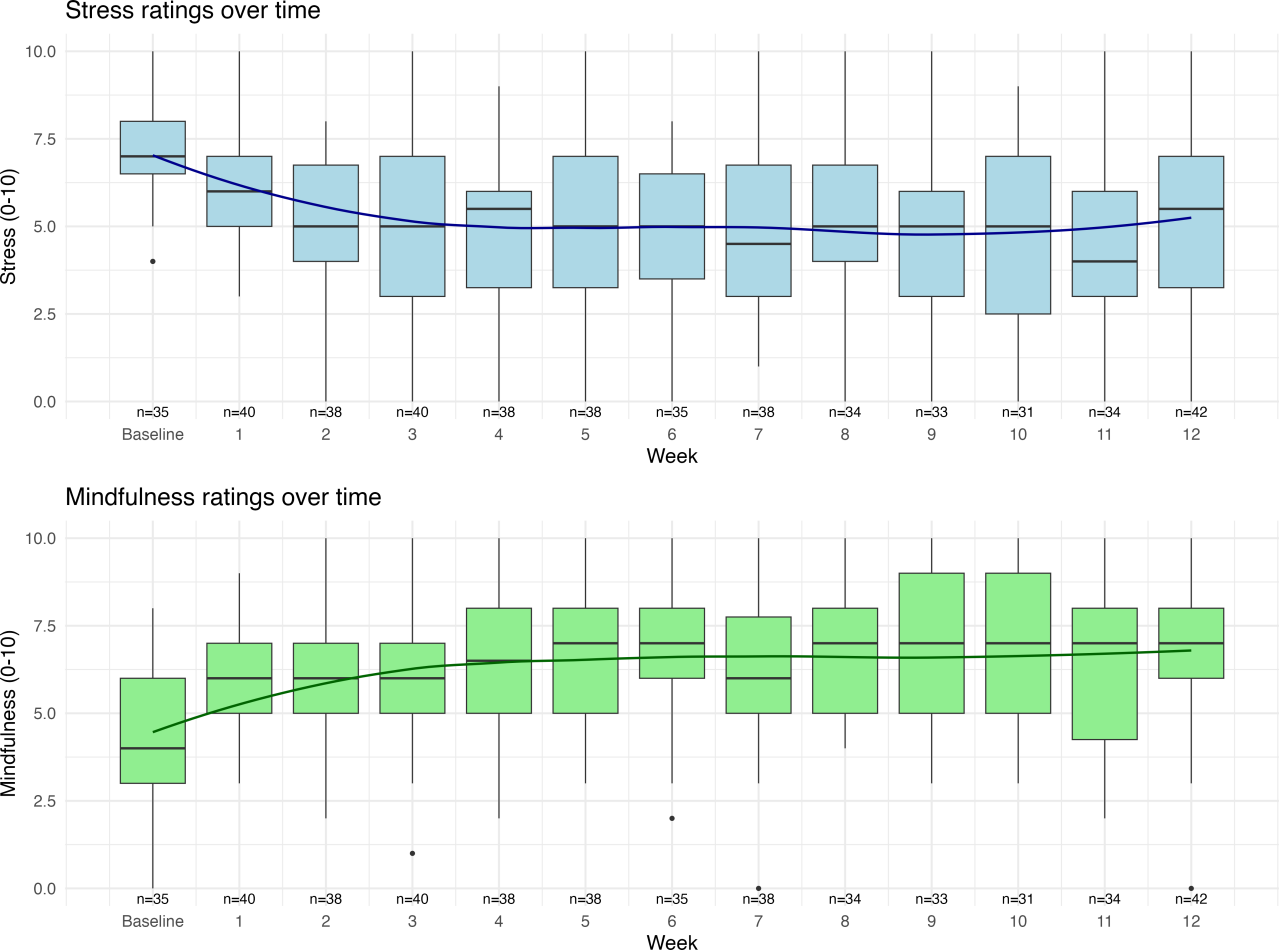
Weekly stress and mindfulness ratings among caregivers of persons living with dementia using the Healthy Minds Program for caregivers.

Median mindfulness and stress ratings at baseline were 4 (IQR 3-6, range 0‐8) and 7 (IQR 6.5-8, range 4‐10), respectively (Figure 1). By week 6 (ie, midway through the study), median mindfulness ratings increased to 7 (range 2‐10) and stress ratings decreased to 5 (range 0‐8). This represents a net increase in median mindfulness of 3 points and a net decrease in median stress of 2 points between baseline and week 6. Mindfulness and stress ratings remained similar throughout the remainder of the trial, with a net increase in median mindfulness and stress of 1 point between week 6 and 12 of the trial. Overall, there was an increase in median mindfulness of 3 points and a decrease in median stress of 1.5 points across the full trial (ie, baseline to week 12).

According to Figure 1, stress ratings appear to have the greatest decrease between baseline and week 3. By week 3, the median stress rating was 5 (IQR 4, range 0‐10), which corresponds to a 2-point decrease from baseline. Mindfulness appears to experience the greatest increase between baseline and week 4. By week 4, the median mindfulness rating was 6.5 (IQR 3, range 2‐10), which corresponds to a 2.5-point increase from baseline.

### Aim 2

**Figure 2.**
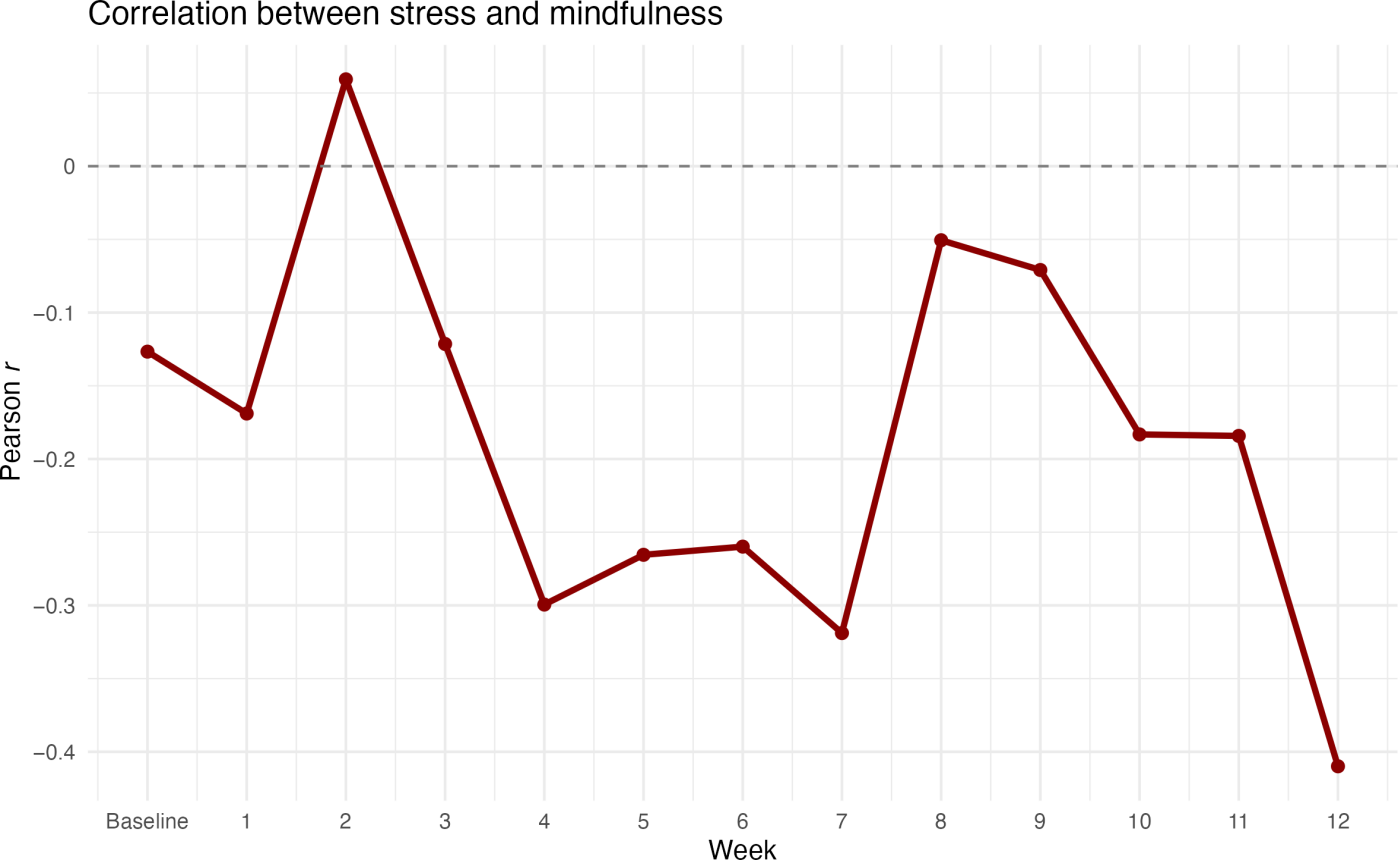
Pearson correlation between mindfulness and stress ratings over time among caregivers of persons living with dementia using Healthy Minds Program for Caregivers.

Figure 2 demonstrates the variability in the correlation between weekly mindfulness and stress ratings. Table 2 reports the results of the GLMM characterizing the relationship between mindfulness and stress. There was a significant fixed effect of mindfulness on stress, such that every additional point of mindfulness above the mean at baseline was correlated with a decrease in participant stress by an average of 0.5 points (*P*<.001). Similarly, participant stress was reduced by 0.06 (*P*=.007), on average, for each week throughout the duration of the study. However, the significant interaction between mindfulness and study week (β=.05, *P*=.001) showed that this association attenuated over time (ie, the stress-reducing effect of mindfulness was most potent during the early weeks of the trial and less potent during the later weeks in the trial). Random effects showed variability in baseline stress (τ_00_=2.04) and the association between mindfulness and stress (τ₁₁=0.08) among participants. Notably, there was a high correlation (ρ_01_=0.86) between intercepts and slopes, meaning that participants with higher baseline stress had a higher association between mindfulness and stress reduction over the course of the trial. Simulation results for the interaction term in this model show approximately 88.80% (95% CI 86.70-90.70) power to detect effects.

**Table 2.** Longitudinal effect of mindfulness ratings on stress ratings among caregivers of persons living with dementia using Healthy Minds Program for Caregivers (N=42).^a^

Predictors	Estimate (95% CI)	*P* value
(Intercept)	5.56 (5.03 to 6.09)	<.001
Mindfulness rating	−0.52 (−0.72 to −0.32)	<.001
Study week	−0.06 (−0.11 to −0.02)	.007
Mindfulness rating × study week	0.05 (0.02 to 0.08)	.001

a Random effects: σ²=2.77; τ₀₀=2.04; τ₁₁=0.08; ρ₀₁=0.86; intraclass correlation coefficient=0.44.

### Aim 3

**Figure 3.**
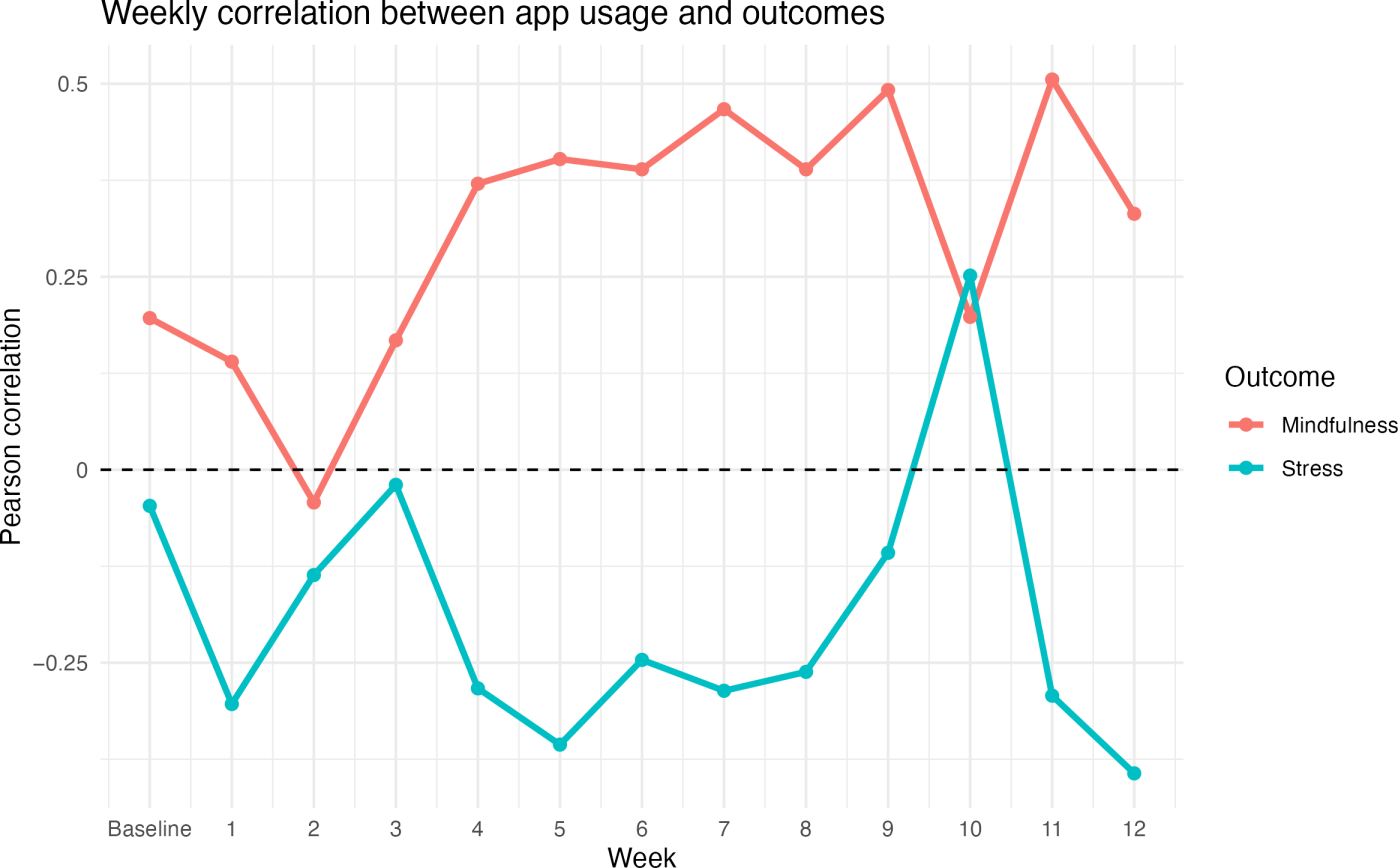
Pearson correlation between Healthy Minds Program for Caregivers (HMP-C) utilization and mindfulness or stress ratings over time among caregivers of persons living with dementia using HMP-C.

Figure 3 details the variability in the correlation between HMP-C utilization and stress and mindfulness ratings each week through the 12-week trial. Correlations largely reflect an inverse relationship, where utilization generally correlates with higher mindfulness and lower stress. This is reflected in Table 3, where the fixed effects of utilization on mindfulness and stress were both significant. For every additional 10 minutes of HMP-C utilization between baseline and week 1, participants rated their mindfulness as 0.14 points higher on average (*P*<.001). Similarly, for every additional 10 minutes of HMP-C utilization, participants rated their stress as 0.14 points lower on average (*P*<.001). Both models demonstrated an effect of time, where for every week of utilization, participants’ mindfulness increased by 0.10 (*P*<.001) and stress decreased by 0.07 (*P*<.001) on average. Both models had statistically significant interaction terms with study week. However, the effect sizes of these interaction terms were very small for both mindfulness (β=.02, *P*<.001) and stress (β=.01, *P*=.01). This suggests that while there is a statistically significant attenuation of the impact of HMP-C utilization on stress and mindfulness ratings, the size of that impact was very small, meaning that this correlation was mostly stable throughout the trial. Simulation results for the interaction term show approximately 99.50% (95% CI 98.84-99.84) power and 68.90% (95% CI 65.93-71.76) power in the mindfulness and stress models, respectively.

**Table 3.** Longitudinal effect of Healthy Minds Program for Caregivers (HMP-C) utilization on weekly mindfulness and stress ratings among caregivers of persons living with dementia using HMP-C (N=42).

Predictors	Mindfulness ratings^a^		Stress ratings^b^	
	Estimates (95% CI)	*P* value	Estimates (95% CI)	*P* value
(Intercept)	5.73 (5.22 to 6.23)	<.001	5.62 (5.11 to 6.13)	<.001
HMP-C utilization^c^	0.14 (0.10 to 0.18)	<.001	−0.14 (−0.20 to −0.08)	<.001
Study week^d^	0.10 (0.07 to 0.13)	<.001	−0.07 (−0.12 to −0.03)	.001
HMP-C utilization × study week	−0.02 (−0.02 to −0.01)	<.001	0.01 (0.00 to 0.02)	.01

a For the mindfulness ratings model, random effects were σ²=1.51, τ₀₀=2.39, intraclass correlation coefficient=0.61, and N=42.

b For the stress ratings model, random effects were σ²=3.00, τ₀₀=2.03, intraclass correlation coefficient=0.40, and N=42.

c HMP-C utilization was mean-centered and scaled to show the effect per 10 additional minutes.

d Study week was centered at the first week after baseline because HMP-C utilization captures past week utilization.

## Discussion

### Principal Results

This secondary data analysis aimed to characterize weekly mindfulness and stress ratings among caregivers of persons living with dementia and explore how digital MBI utilization was associated with those ratings throughout 12 weeks of a feasibility trial. We found that, on average, participants’ mindfulness and stress ratings improved throughout the initial weeks of the study and then sustained that improvement throughout the remainder of the study duration. Further, we found that there was a significant association between mindfulness and stress ratings throughout the study duration among our sample of caregivers of persons living with dementia, such that in weeks when mindfulness was high, stress was low. However, this association became weaker as the study progressed, suggesting that perceived stress and mindfulness are less intertwined over time. Finally, HMP-C utilization was associated with changes in mindfulness and stress ratings, suggesting that continued utilization may be helpful for maintaining these improvements over time.

The sample of participants from this study in some ways reflects the broader population of caregivers of persons living with dementia and in other ways potentially deviates from the average caregiver of persons living with dementia. On the one hand, participants overwhelmingly identified as women. Women have historically undertaken the role of caregiving for persons living with dementia, and this finding is consistent across geographic regions. Further, women often spend more time caregiving than their male caregiver counterparts and often experience a higher level of caregiver burden [36]. Conversely, the present sample of participants is largely White, has attained a graduate degree, and is earning more than US $100,000 per year. Past work has shown that approximately two-thirds of caregivers of persons living with dementia are White (compared to roughly 4 in 5 caregivers in the present sample). Further, past work has shown that the average caregiver of persons living with dementia earns US $50,000 or less per year. Additionally, the present sample represents a cohort of individuals positioned to have greater access to and literacy with technology due to their demographic characteristics (eg, race, income, education, and age) [37]. The complex intersectionality of underresourced identities and technological access and literacy can impact digital intervention engagement. Indeed, past reviews have shown that technological literacy is a significant barrier to implementing digital interventions for caregivers of persons living with dementia, despite their high health literacy [38-44].

Both mindfulness and stress ratings improved throughout the first 6 weeks of the study, with most of the improvement occurring between the first 3 to 4 weeks. From there, mindfulness and stress ratings leveled off throughout the remainder of the study. These findings suggest that participants may have developed some level of mastery in mindfulness skills within the first 4 weeks of HMP-C utilization, with more minor improvements occurring after week 4. Since Dahl et al [8] conceptualize mindfulness practice through a series of developable skills, it would be expected that once those skills are mastered, continued practice would lead to maintenance (ie, not a linear increase). This study reflects this trajectory, where caregivers improved mindfulness and stress early (weeks 1‐4) and then stabilized by week 6. This may reflect acquisition of new skills followed by subsequent consolidation of those skills. It remains plausible that continued practice would yield additional benefit at longer follow-up. Future work should evaluate this by extending assessments over a longer period of time.

Improvements in weekly mindfulness ratings correlated with stress reductions throughout the study duration. However, there is context to this finding. First, there was variability in baseline stress and the association between mindfulness and stress across participants. Notably, there was a positive correlation between baseline stress and the effect of mindfulness on stress reductions, meaning those who were the most stressed at baseline benefited the most from increases in mindfulness. This suggests that mindfulness skills may have offered these participants a strategy to immediately manage stressful situations in their lives. However, the association between mindfulness and stress decreased over time, regardless of baseline stress levels. This trajectory in mindfulness and stress ratings reflects several previous findings, where an initial effect of mindfulness skills on the primary outcome is observed, followed by a period of attenuation [45-48]. These findings underscore the importance of considering both individual differences at baseline and the temporal dynamics of mindfulness practice. The early pronounced association for those with higher stress highlights mindfulness as a potentially powerful mechanism for alleviating acute distress (ie, state mindfulness for coping). At the same time, the subsequent attenuation of effects suggests that the role of mindfulness may shift from producing large, immediate reductions in stress to supporting the maintenance of stress reduction over time (ie, trait mindfulness). This pattern is consistent with theoretical models, such as Dahl et al [8], that view mindfulness as a set of skills that, once acquired, foster a sense of resiliency and stability, rather than indefinite linear improvement. Taken together, these results suggest that digital MBIs should emphasize intensive early engagement while also providing education and strategies to sustain and deepen benefits during later phases of practice. Future research should aim to assess the mediating role of mindfulness in stress reduction for caregivers of persons living with dementia as compared to an attention-placebo control condition in a fully powered efficacy trial.

HMP-C utilization was broadly associated with increases in mindfulness and reductions in stress ratings throughout the duration of the study. Despite a statistically significant interaction of HMP-C utilization and time in study weeks in both utilization models, the effect sizes of these interactions were very small. While there may be some minor attenuation of the association between HMP-C utilization on mindfulness and stress over time, the association was largely maintained throughout the study duration. This suggests that continued utilization of HMP-C may serve as an important tool for mindfulness skill maintenance, such that utilization may be protective against returning to baseline levels of mindfulness and stress. Therefore, continued use of the digital MBI would be recommended for achieving and sustaining improvements in mindfulness and stress among caregivers of persons living with dementia. These findings suggest several directions for future work. First, to examine if and to what extent a dose-response relationship exists between HMP-C utilization and mindfulness or stress, a future study could randomize participants to incrementally different “dosages” of HMP-C utilization (eg, 45, 60, 75, and 90 min/wk). Further, future research is needed to address whether mindfulness skills and their effect on stress can be maintained without continued practice or the same amount of continued practice. For example, future studies may choose to randomize participants to continued, reduced, or no HMP-C utilization after an initial period of 4 weeks. These would provide the rigorous groundwork needed to tune digital MBI utilization recommendations.

### Limitations

There are several limitations to consider when interpreting the findings from this study. First, the participants in this study were a small sample from a stage 1b feasibility RCT. In many ways, the sample was not representative of caregivers of persons living with dementia broadly with respect to race, income, education, and potentially digital literacy. Due to this, the generalizability of these findings to the broader population of caregivers may be limited. Further, analytically, we were not powered to control for possible sources of confounding, such as digital and health-related literacy, which may have led participants to be more adept at improving from mindfulness skill practice. Second, this study does not include a control or comparator arm in the analysis. This precludes making causal statements about the efficacy of HMP-C or digital MBIs with respect to changes in mindfulness and stress. Possible sources of confounding due to lack of randomization include seasonal changes, regression toward the mean, or simply random fluctuations. Third, single-item weekly ratings are less explanatory than more comprehensive measures. For example, from this study, we cannot ascertain exactly what stressors participants were experiencing or were being mitigated. However, single-item measures have been shown to be comparable to full-scale measures as broad measures of stress or mindfulness, suggesting that the overall trends observed are likely valid [49-51]. Fourth, week-over-week changes in mindfulness and stress may be reflective of the Hawthorne effect or assessment reactivity, as opposed to true changes in the measured constructs.

### Conclusions

This study is an exploratory secondary analysis of a feasibility pilot RCT of HMP-C, a digital MBI tailored for caregivers of persons living with dementia. We found that mindfulness and stress ratings improved throughout the first 3 to 4 weeks of the trial before leveling off throughout the remainder of the trial. Particularly, in these first 4 to 6 weeks of skill acquisition, mindfulness and stress were significantly negatively related to one another. This association was higher for those with high baseline stress. Together, these findings highlight the potential benefit of digital MBIs for stress reduction among caregivers of persons living with dementia and suggest that mindfulness skills may be acquired within the first 4 to 6 weeks of the intervention and then maintained over a longer period of time. Future research is needed to move closer to precise dosing recommendations for mobile apps with this population.

## Abbreviations

GLMM: generalized linear mixed model
HMP-C: Healthy Minds Program for Caregivers
MBI: mindfulness-based intervention
RCT: randomized controlled trial
REDCap: Research Electronic Data Capture
